# The Role of SGLT2-Inhibitors Across All Stages of Heart Failure and Mechanisms of Early Clinical Benefit: From Prevention to Advanced Heart Failure

**DOI:** 10.3390/biomedicines13030608

**Published:** 2025-03-03

**Authors:** Simone Pasquale Crispino, Andrea Segreti, Vincenzo Nafisio, Daniele Valente, Filippo Crisci, Aurora Ferro, Ilaria Cavallari, Annunziata Nusca, Gian Paolo Ussia, Francesco Grigioni

**Affiliations:** 1 Department of Cardiovascular Sciences, Fondazione Policlinico Campus Bio-Medico di Roma, 00128 Rome, Italy; simone.crispino@unicampus.it (S.P.C.); vincenzo.nafisio@unicampus.it (V.N.); daniele.valente@unicampus.it (D.V.); filippo.crisci@unicampus.it (F.C.); aurora.ferro@unicampus.it (A.F.); i.cavallari@policlinicocampus.it (I.C.); a.nusca@policlinicocampus.it (A.N.); g.ussia@policlinicocampus.it (G.P.U.); f.grigioni@policlinicocampus.it (F.G.); 2 Department of Movement, Human and Health Sciences, University of Rome “Foro Italico”, 00135 Rome, Italy

**Keywords:** acute heart failure (AHF), chronic heart failure (CHF), four pillars, foundational therapy, GDMT, heart failure (HF), heart failure with improved ejection fraction (HFimpEF), heart failure with preserved ejection fraction (HFpEF), heart failure with reduced ejection fraction (HFrEF), stage D heart failure, sodium-glucose cotransporter-2 inhibitor (SGLT2i)

## Abstract

Sodium-glucose cotransporter-2 inhibitors (SGLT2i), initially developed as antihyperglycemic agents, have revolutionized heart failure (HF) management, offering substantial benefits across all stages and phenotypes of the disease. Regardless of left ventricular ejection fraction (LVEF), these agents have proven efficacy in both chronic and acute HF presentations. This review explores SGLT2i applications spanning the HF continuum, from early stages (Stage A) in at-risk individuals to the mitigation of progression in advanced HF (Stage D). Evidence from numerous trials has shown that SGLT2i significantly lower rates of HF hospitalization, improve renal function, and decreases cardiovascular mortality, highlighting their multifaced mechanisms of action in HF care. This review also highlights the potential mechanisms by which SGLT2i exert their beneficial effects on the cardiovascular and renal systems, each contributing to early and sustained clinical improvements. However, the integration of SGLT2i into guideline-directed medical therapy poses practical challenges, including initiation timing, dosing, and monitoring, which are addressed to support effective treatment adaptation across patient populations. Ultimately, this review provides a comprehensive assessment of SGLT2i as a foundational therapy in HF, emphasizing their role as an intervention across multiple stages aimed at improving outcomes across the entire HF spectrum.

## 1. Introduction

### 1.1. Background

Heart failure (HF) is a clinical syndrome characterized by specific signs and symptoms due to structural and/or functional abnormalities confirmed by elevated natriuretic peptide levels or objective evidence of pulmonary/systemic congestion [1,2]. HF is a global public health problem affecting an estimated 64 million people worldwide, with the 5-year mortality rate being approximately 50% [2,3]. The prevalence of HF ranges from 1% to 3% in the general adult population in industrialized countries and is projected to rise significantly due to improved diagnostic tools that ensure accurate diagnoses and life-saving medical treatments that extend survival post diagnosis [4].

HF is the leading cause of hospitalization in the United States and Europe, resulting in over 1 million admissions as a primary diagnosis and representing 1% to 2% of overall hospitalizations. Annual healthcare costs are estimated to exceed 30 billion dollars [5,6,7]. Each HF episode reflects clinical deterioration and is associated with a significantly increased mortality risk. This high-risk state is a prime target for novel therapies aimed at improving outcomes, despite substantial heterogeneity in pathophysiology and patient characteristics [5]. Each hospitalization for HF (HHF) represents a critical opportunity to optimize the management of pharmacological therapy through the implementation of evidence-based treatments in a controlled setting. Notably, initiating guideline-directed medical therapy (GDMT) during hospitalization has been identified as one of the strongest predictors of long-term treatment adherence [5].

In recent years, HF management has advanced significantly, leveraging a combination of neurohormonal modulators and device therapies. The four pillars of treatment for HF with reduced ejection fraction (HFrEF) now include beta-blockers, mineralocorticoid receptor antagonists (MRAs), sodium-glucose cotransporter-2 inhibitors (SGLT2i), and either angiotensin-converting enzyme inhibitors (ACEi), angiotensin receptor–neprilysin inhibitors (ARNIs), or angiotensin receptor blockers (ARBs) [8]. The early benefits of GDMT support prompt initiation, with goals that include reducing mortality; preventing recurrent hospitalizations; and improving clinical status, functional capacity, and quality of life (QoL) [1,9,10].

A significant update in the 2023 European Society of Cardiology (ESC) guidelines is the Class I, Level A recommendation for SGLT2i in patients with HF with preserved (HFpEF) and mildly reduced ejection fraction (HFmrEF) [8]. SGLT2i have demonstrated a pivotal role in preventing and treating HF across the entire spectrum of left ventricular ejection fraction (LVEF), regardless of the presence of type 2 diabetes (T2D) or chronic kidney disease (CKD) [11,12]. Randomized trials enrolling HF patients with varying LVEF have confirmed the efficacy of dapagliflozin and empagliflozin, in combination with conventional therapies, in reducing the risk of cardiovascular death and HHF [11].

Post hoc and prespecified analyses from major trials indicate an early clinical benefit of SGLT2i treatment, observable as soon as two weeks after initiation. However, the mechanisms underlying these rapid benefits are not yet fully understood [11,13]. Furthermore, clinical trials involving patients recently hospitalized for worsening congestion have shown substantial symptomatic improvement compared to placebo, as evidenced by improved Kansas City Cardiomyopathy Questionnaire (KCCQ) scores among patients with severe symptoms [3].

### 1.2. Classifications and Phenotyping

Over the last decade, HF has been traditionally classified into three primary phenotypes based on left ventricular ejection fraction (LVEF): reduced (HFrEF), mildly reduced (HFmrEF), and preserved (HFpEF). Most recently, the DELIVER trial shed light on an additional phenotype, HF with improved EF (HFimpEF), which encompasses patients previously diagnosed with HFrEF who now present with an improved LVEF (>40%), while maintaining the indication for HFrEF-targeted therapy [4]. This classification was initially adopted because clinical trial inclusion criteria, and the resulting evidence of therapeutic benefit, were primarily limited to patients with reduced LVEF.

Over time, the reliance on LVEF classification has persisted due to its therapeutic and prognostic value [14]. However, categorizing HF solely based on LVEF risks oversimplifying an inherently complex condition, as multiple overlapping mechanisms often underlie HF across phenotypes [15].

In outpatient settings, data from the European Society of Cardiology (ESC) Heart Failure Long-Term Registry, which included 9134 outpatients with HF over a one-year follow-up, revealed that 60% of patients presented with HFrEF, 24% with HFmrEF, and 16% with HFpEF [16].

Despite these classifications, HF is a heterogeneous syndrome, and its complexity manifests in the diverse etiologies and overlapping pathophysiological mechanisms observed across phenotypes.

Beyond LVEF-based phenotypes, additional classifications exist. The New York Heart Association (NYHA) functional classification categorizes HF based on symptom burden, specifically dyspnea, and serves as a tool to assess quality of life. It also aids decision making for advanced therapies, such as transplantation or device therapy, in severe cases.

More recently, the universal definition of HF [2] introduced a structural progression framework, delineating four stages of HF (from A to D) adding a structural progression perspective, delineating HF from risk factors alone (Stage A) through asymptomatic (stage B), symptomatic HF (Stage C) to advanced, treatment-refractory cases (Stage D).

For late-stage HF patients, INTERMACS (Interagency Registry for Mechanically Assisted Circulatory Support) profiles offer seven categories, evaluating hemodynamic stability and inotropic support requirements [17,18]. These profiles guide the timing of mechanical circulatory assist devices.

In the 2023 ESC guidelines update [19], the task force considered adopting a revised classification system, including HFnEF (Heart Failure with normal ejection fraction) and adjusted LVEF thresholds for HFrEF (i.e., <45%). However, due to insufficient consensus, further changes in terminology were deferred to the next guideline update.

In summary, while LVEF remains a critical tool for phenotyping HF, it is essential to integrate other dimensions, such as structural progression and symptom burden, to account for the complexity and individualized therapeutic needs of HF across its continuum. Table 1 and Table 2 summarize actual definitions and phenotypes of HF.

#### 1.2.1. HFrEF

This category is primarily characterized by ischemic heart disease and dilated idiopathic cardiomyopathy as its leading causes, while less common etiologies include hypertension, valvular heart disease, and arrhythmias [20,21]. From a pathophysiological standpoint, HFrEF involves excessive activation of the neurohormonal axis, particularly the sympathetic nervous system and the renin–angiotensin–aldosterone system (RAAS). Initially adaptive, these mechanisms become pathological over time, leading to salt and water retention and adverse hemodynamic effects [22].

In recent decades, pharmacological antagonism of these pathways, using landmark therapies such as beta-blockers, MRA, ACEi, and ARBs, has been proven to significantly reduce morbidity and mortality. More recently, innovative treatments such as ARNIs and SGLT2i have been shown to improve major cardiovascular outcomes in HF patients, irrespective of LVEF [23].

Patients with HFrEF face a high risk of mortality, primarily due to progressive HF or sudden arrhythmias. Factors influencing sudden cardiac death (SCD) include LVEF, ischemic etiology of HF, the presence of scar or late gadolinium enhancement (LGE) on cardiac magnetic resonance (CMR) [24], as well as the patient’s age and sex. To mitigate these risks, therapies such as an implantable cardioverter defibrillator (ICD) or cardiac resynchronization therapy (CRT) may be considered. Diuretics also play an essential role, providing symptom relief by alleviating congestion and improving quality of life, although not improving prognosis [1].

#### 1.2.2. HFpEF and HFmrEF

With an increasing prevalence, HF patients with higher LVEF (>40%) now represent a significant proportion of the HF population. According to the previously cited ESC registry, 24% of patients present with HFmrEF and 16% with HFpEF among the overall HF population [5]. HFmrEF patients often share clinical features and risk factors more similar to those with HFrEF than HFpEF. For instance, HFmrEF patients are frequently male, younger, and exhibit a higher incidence of coronary artery disease (CAD), but present with lower rates of atrial fibrillation (AF) and fewer noncardiac comorbidities [25]. Despite these traits, the mortality rate in HFmrEF is generally lower than HFrEF and approximates that of HFpEF [1].

HFpEF patients, on the other hand, are often older, predominantly female, and exhibit higher rates of atrial fibrillation, chronic kidney disease (CKD), obesity, metabolic dysfunction, sedentary lifestyle, and other non-cardiovascular comorbidities compared to HFrEF patients [1,26,27].

This suggests a pathogenetic role for systemic inflammation, endothelial dysfunction, and altered energy metabolism, positioning HFpEF as a systemic syndrome characterized by “reserve dysfunction”, which affects multiple organs beyond the heart [25].

Although SGLT2i have demonstrated positive results in HFpEF, the DELIVER and EMPEROR-Preserved studies showed an absolute risk reduction of only 3% for the primary endpoint, which was mainly driven by reductions in HHF rather than cardiovascular mortality. These findings may underscore the need for enhanced diagnostic tools and tailored treatments for HFpEF based on phenotyping [25].

#### 1.2.3. HFimpEF

It is essential to recognize and classify patients whose LVEF improves after initial reduction. These patients, referred to as having HFrEF with recovered systolic function or HF with improved LVEF (HFimpEF) [1], exhibit a better prognosis compared to those with persistent HFrEF.

HFimpEF patients often share common characteristics, including being younger, having fewer clinical comorbidities, and demonstrating a lower left ventricular end-diastolic diameter (LVEDD) [14]. These individuals continue to meet the criteria for HFrEF therapy, emphasizing the importance of ongoing treatment despite LVEF recovery.

## 2. Methods

This narrative review provides a comprehensive analysis of the use of SGLT2i across all stages of HF treatment. A systematic literature search was conducted using PubMed, Scopus, and Web of Science to identify relevant studies published up to December 2024. We included randomized controlled trials (RCTs), meta-analyses, observational cohort studies, expert consensus statements, and guideline recommendations from major cardiology societies. Case reports, small-scale studies with insufficient statistical power, and non-English articles were excluded. Two authors independently screened articles by reviewing titles and abstracts, and full-text articles were assessed based on their relevance to the clinical use, mechanisms of action, and safety profile of SGLT2i in HF. Reference lists of selected papers were also examined to identify additional relevant studies. By adopting this structured approach, the review aims to provide an updated and accessible resource that underscores the potential of SGLT2i in HF management, integrating evidence across different HF phenotypes and stages.

## 3. SGLT2-Inhibitors Across the Trajectory of Heart Failure

Initially introduced as antihyperglycemic drugs [28], SGLT2i have demonstrated significant cardiovascular benefits, particularly in the setting of chronic stable HF [29].

The DAPA-HF and the EMPEROR-REDUCED trials were the first large-scale trials exploring their effects on cardiac protection and their efficacy in reducing hospitalization and mortality in HF patients [30,31]. The DAPA-HF trial investigated the long-term effects of dapagliflozin in patients with HFrEF compared to placebo on top of optimal medical therapy: a reduction in hospitalization and CV death by 26% was observed [31]. The EMPEROR-REDUCED trial explored the effects of empagliflozin in the same setting of patients: a 25% reduction rate in HF worsening and all-cause mortality was observed [30]. Empagliflozin has also demonstrated to be effective in delaying estimated glomerular filtration rate (eGFR) decline in CKD patients.

Given these robust outcomes, SGLT2i were integrated as first-line therapy for HFrEF in the 2021 ESC guidelines for the diagnosis and treatment of acute and chronic HF, receiving a Class IA recommendation [1]. Subsequent findings from two major trials, the EMPEROR-Preserved and DELIVER studies, extended the clinical indications of SGLT2i to include HF with mildly reduced ejection fraction (HFmrEF) and HF with preserved ejection fraction (HFpEF) [32,33]. As per the 2023 ESC Focus Update, dapagliflozin and empagliflozin are now recommended with the same Class IA designation for HFmrEF and HFpEF management [34]. 

A meta-analysis encompassing six pivotal SGLT2-inhibitor trials, EMPA-REG OUTCOME, CANVAS, DECLARE-TIMI 58, CREDENCE, VERTIS CV, and SCORED, demonstrated a significant reduction in the time to the first event of major adverse cardiovascular events (MACEs), encompassing CV death, myocardial infarction (MI), and stroke, across diverse HF clinical stages [35].

Several studies underscore the importance of early initiation of SGLT2i following HF diagnosis, as their benefits manifest rapidly after commencement [36,37,38]. It is strongly recommended that SGLT2i be initiated promptly in patients hospitalized for AHF, provided no contraindications exist, and that therapy be continued post discharge [39]. These agents are not only well-tolerated, but also facilitate decongestion without impairing renal function, while delivering comprehensive CV benefits [39].

In defining the universal stages of HF, Bozkurt et al. delineated four phenotypes, ranging from those at risk for HF but without current or prior symptoms (Stage A) to those with advanced symptoms requiring recurrent hospitalization (Stage D) [2].

Table 2 contains a brief description of each stage as stated in the universal definition of HF [40].

**Table 2:** Stages of HF, according to Universal Definition [40].

Heart Failure Stage	Definition	Clinical Implications
Stage A HF (At Risk)	No structural heart disease, but high-risk conditions present (e.g., hypertension, diabetes, obesity, CKD).	Risk factor modification, lifestyle interventions, SGLT2i in diabetics to prevent HF, or hypertensive medications in affected patients. Early intervention prevent progression.
Stage B HF (Preclinical HF)	Structural heart disease but no symptoms.	Early initiation of HF therapies to prevent progression as recommended for each phenotype.
Stage C HF (Symptomatic HF)	Current or past HF symptoms with structural disease.	GDMT initiation (Beta-blockers, ACEi/ARB/ARNI, MRA, SGLT2i), diuretics for symptom relief, device therapy in select cases. Regular monitoring required to avoid worsening HF.
Stage D HF (Advanced HF)	Refractory HF symptoms despite GDMT, requiring specialized interventions.	Consideration of heart transplant, LVAD, palliative care. High-mortality risk, requires advanced HF management.

Abbreviations: ACEi: Angiotensin-Converting Enzyme Inhibitor; ARB: Angiotensin II Receptor Blocker; ARNI: Angiotensin Receptor-Neprilysin Inhibitor; CKD: Chronic Kidney Disease; GDMT: Guideline-Directed Medical Therapy; HF: Heart Failure; LVAD: Left Ventricular Assist Device; MRA: Mineralocorticoid Receptor Antagonist; SGLT2i: Sodium-Glucose Cotransporter-2 Inhibitors.

### 3.1. Stage A: Prevention

Patients categorized as Stage A are those at risk of developing HF due to the presence of risk factors, comorbidities, known exposure to cardiotoxins, or a family history of cardiomyopathy. Additionally, patients with evidence of structural heart disease, abnormal cardiac function, or elevated natriuretic peptide levels, despite the absence of HF symptoms or signs, face an increased risk of all-cause mortality and hospitalization [41,42]. T2D is particularly common in individuals at elevated risk of CVD. SGLT2i such as empagliflozin and canagliflozin have demonstrated reductions in the risk of MACE, with all agents in this class showing efficacy in reducing HF incidence and CKD progression [35,43].

The EMPA-REG-OUTCOME trial, a randomized placebo-controlled study, evaluated the effects of empagliflozin in addition to standard care on cardiovascular morbidity and mortality in patients with T2D and high CV risk. Among 7020 participants, no significant differences were observed in rates of myocardial infarction or stroke between groups; however, the empagliflozin cohort experienced significantly lower rates of CV death (38% risk reduction), 35% of HHF risk reduction, and all-cause mortality (32% risk reduction) without significant increases in adverse events [44].

The CANVAS trial assessed the cardiovascular, renal, and safety outcomes of canagliflozin in a cohort of 10,142 participants with T2D and high CV risk. The trial reported lower rates of CV death, nonfatal myocardial infarction, and nonfatal stroke with canagliflozin compared to placebo (hazard ratio [HR] 0.86) [45].

The DECLARE-TIMI 58 trial evaluated dapagliflozin in patients with T2D who had or were at risk for atherosclerotic cardiovascular disease (ASCVD). Although dapagliflozin did not reduce the overall MACE rate, it was associated with a 17% relative risk reduction in CV death or HHF and a 24% risk reduction in renal events compared to placebo [46].

In the CREDENCE trial, patients with T2D and CKD were randomized to canagliflozin or placebo. Canagliflozin demonstrated a 30% risk reduction in death from renal or cardiovascular causes and a 39% reduction in HHF [47].

The VERTIS CV trial studied ertugliflozin in 8246 patients with T2D and ASCVD, revealing no significant differences in MACE rates between groups but a lower rate of HHF in the ertugliflozin arm compared to placebo (8.1% vs. 9.1%) [48].

The SCORED trial explored sotagliflozin in diabetic patients with CKD. Participants receiving sotagliflozin experienced reduced rates of CV death and HHF compared to placebo; however, the intervention group reported higher rates of adverse events [38].

In light of these findings, the 2023 ESC guidelines for the management of cardiovascular disease in patients with diabetes recommend the use of SGLT2i to reduce HHF in patients with T2D at risk of HF or CKD, irrespective of glucose control, HbA1c levels, or concurrent glucose-lowering therapies (Class 1A) [49].

In conclusion, the accumulating evidence underscores the importance of addressing individual risk factors in patients at risk of HF, particularly those with T2D and CKD. In this context, SGLT2i have demonstrated remarkable efficacy in reducing HHF and mortality across diverse patient populations, regardless of glycemic control or renal function. This comprehensive benefit highlights the potential of SGLT2i to slow disease progression in high-risk individuals, emphasizing a shift toward a holistic cardiovascular protection strategy, akin to established preventive measures in coronary syndromes. Main evidence presented in this paragraph is summarized in Table 3.

### 3.2. Stage B and C: Preclinical and Clinical HF

Stage B represents the preclinical phase of HF, where structural heart disease is evident but symptoms are absent [2]. Common causes include prior myocardial infarction, valvular heart disease, or cardiomyopathies. Despite the lack of symptoms, these patients are at significantly higher risk for progression to symptomatic HF (Stage C) and are prone to adverse CV events, including hospitalization and mortality. In contrast, Stage C encompasses patients with current or prior HF symptoms due to structural or functional cardiac abnormalities [2].

The benefits of SGLT2i utilization are well established in these stages, regardless of LVEF [11]. Large clinical trials prompted updates to the ESC Guidelines, which now recommend dapagliflozin or empagliflozin to reduce HHF and CV death, irrespective of LVEF [1,19].

The first two randomized trials to investigate the effect of an SGLT2i compared with placebo in patients with HFrEF with and without T2D were the DAPA-HF [31] and the EMPEROR-Reduced [30]. The former included patients NYHA class II–IV, with LVEF ≤ 40% despite OMT, and elevated NT-proBNP, and randomly assigned in a 1:1 ratio to receive dapagliflozin 10 mg or placebo on top of optimal medical therapy: a 26% risk reduction rate of worsening HF and CV death was observed, and, in addition, dapagliflozin did improve symptoms, physical function, and quality of life [31]. The EMPEROR-REDUCED Trial one evaluated empagliflozin vs. placebo within HFrEF patients, in NYHA class II–IV, and LVEF ≤ 40% despite OMT, and an elevated NT-proBNP. Empagliflozin was able to reduce the risk of CV death or HHF by 25% and to improve quality of life. This effect was consistent across patients with and without diabetes at baseline [30].

The EMPEROR-Preserved trial was the first large-scale study to evaluate patients with HFpEF. It included approximately 6000 patients with HF in NYHA class II–IV, LVEF > 40%, and elevated serum NT-proBNP levels, randomized to receive Empagliflozin or placebo. The trial demonstrated a significant reduction in the primary composite endpoint of worsening HF and cardiovascular (CV) death, with a hazard ratio (HR) of 0.79 [30].

Similarly, the DELIVER trial assessed the effects of dapagliflozin in HF patients with NYHA class II–IV and LVEF > 40%, including those with prior LVEF < 40%. Dapagliflozin reduced the primary endpoint of CV death or worsening HF by 18% compared to placebo [40]. These findings establish the efficacy of SGLT2-inhibitors (SGLT2i) across all HF settings, regardless of LVEF or diabetes status [50]. SGLT2i have shown clear benefits in all settings of HF, regardless of LVEF and the presence of T2D.

### 3.3. Acute HF

Evidence is mounting regarding the benefits of SGLT2i in acute HF (AHF), particularly in enhancing early decongestion when used synergistically with loop diuretics [49]. The timing of SGLT2i initiation in AHF, however, remains a topic of ongoing discussion.

The EMPULSE Trial was designed to evaluate the effects of empagliflozin on the improvement of survival and symptoms in patients hospitalized for AHF [51]. Patients were randomized approximately three days after admission to receive empagliflozin or placebo. Early initiation of empagliflozin led to a significant reduction in all-cause mortality and improved quality of life at 90 days. The results indicated improved diuretic efficiency and earlier decongestion, consistent across all LVEF ranges and in both acute de novo and decompensated chronic HF. Mortality was lower in the empagliflozin group (4.2%) compared to the placebo group (8.3%) [51]. The EMPA-RESPONSE-AHF trial, a double-blind, placebo-controlled pilot study, evaluated empagliflozin in AHF patients within 24 h of admission. A post hoc analysis revealed that empagliflozin enhanced urinary output and achieved a more negative fluid balance when used in conjunction with loop diuretics. Importantly, these effects were observed without significant alterations in plasma glucose levels, even in non-diabetic patients [36]. A post hoc analysis showed that SGLT2i had a synergistic effect, with loop diuretics leading to a higher urinary output and a more negative fluid balance in those treated with empagliflozin when compared with placebo. Moreover, urinary glucose excretion was similar in patients with and without diabetes, and plasma glucose levels remained unchanged [36].

The EMPAG-HF study aimed to evaluate whether early initiation of SGLT2 inhibition with empagliflozin, in combination with standard medical therapy, could enhance diuresis without exacerbating kidney injury in patients admitted with AHF within the first 12 h. The study monitored patients during the critical early phase of AHF and assessed outcomes such as cumulative urine output over five days, increases in creatinine levels (>0.3 mg/dL), and changes in baseline kidney function. Results indicated that early empagliflozin initiation led to a 25% increase in cumulative urine output over five days, improved diuretic efficiency, and a more pronounced reduction in NT-proBNP levels [52]. However, the evidence supporting SGLT2i’s decongestive effects in AHF remains limited [37]. Nonetheless, real-world data suggest a rapid increase in the prescription rates of SGLT2i during AHF hospitalizations [53]. Early initiation during hospitalization has been significantly associated with a lower risk of adverse composite events post discharge. These benefits have been observed consistently across patients with AHF, irrespective of LVEF status [53].

### 3.4. Worsening HF

Worsening heart failure represents a critical phase in the progression of chronic HF, characterized by episodes of intensifying symptoms and signs that may necessitate interventions ranging from outpatient intravenous diuretic therapy to full hospitalization [54,55]. WHF is recognized as a pivotal event in HF’s trajectory, marking a transition to a more severe state and often associated with significantly elevated post-discharge mortality and readmission rates. Recently, following positive outcomes from the VICTORIA trial [56], evaluating the vericiguat in this setting, both the ACC [54] and the ESC [55], highlighted WHF as a distinct and clinically important phase in HF, characterized by a marked increase in adverse outcomes. Nowadays, WHF management focuses on decongestion, rapid optimization of GDMT, and addressing precipitating factors, underscoring the urgency of intervention during these episodes.

Regarding SGLT2i in WHF, the SOLOIST-WHF trial provides the most relevant large-scale evidence. This study evaluated sotagliflozin in diabetic patients experiencing WHF, with therapy initiated either prior to or shortly after hospital discharge. Sotagliflozin demonstrated a substantial reduction in cardiovascular deaths and HF hospitalizations (HR 0.67), although the trial did not analyze its specific decongestive effects [38]. No large-scale trials data exist on the two approved medications for HF: dapagliflozin and empagliflozin. Table 4 summarizes main clinical trial data on WHF.

### 3.5. Stage D: Advanced HF

Advanced HF (AdHF) represents the terminal stage of the disease, where patients experience persistent, severe symptoms that remain unresponsive to optimal medical therapy [16]. This phase is characterized by significant morbidity and mortality, as conventional pharmacological and device-based therapies are often inadequate to manage AdHF effectively. Advanced interventions, including heart transplantation or long-term mechanical circulatory support (MCS), represent the primary options, yet these are only viable for a limited subset of patients meeting specific eligibility criteria. Moreover, data from large-scale randomized clinical trials focused on the management of AdHF remain scarce, creating substantial gaps in evidence-based recommendations. The Heart Failure Association (HFA) of the ESC [57] underscores that in the context of AdHF, clinical objectives shift from modifying disease progression to prioritizing symptom relief and enhancing QoL. For most patients at this stage, reversing disease progression is no longer a realistic goal, necessitating a palliative approach that addresses both physical and psychosocial needs.

In light of these challenges, preventive strategies aimed at decelerating the progression of HF are critical. Early identification and intervention in at-risk populations, optimized treatment during the earlier stages of HF, and effective management of contributing risk factors can collectively reduce the likelihood of progression to AdHF [58]. This prevention-focused paradigm aligns with the growing emphasis on comprehensive risk reduction and personalized care.

Despite the limited evidence specific to AdHF, the benefits of SGLT2i across various stages of HF may suggest a role in delaying the transition to advanced stages. These insights highlight the importance of timely and proactive management throughout the HF continuum, underscoring the need for continued research and evidence-based strategies to address the challenges of advanced HF.

**Figure 1:**
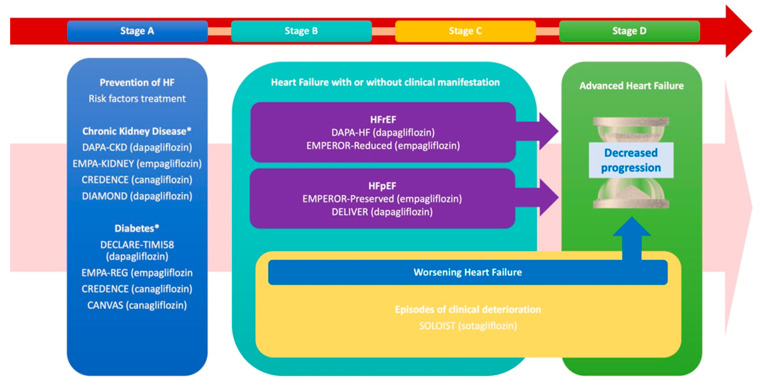
The progression and management of heart failure (HF) across its clinical stages (A–D), emphasizing targeted interventions for risk reduction, prevention of worsening events, and progression to advanced HF, while referencing key clinical trials. Stage A prioritizes HF prevention through the management of risk factors such as CKD and T2D, supported by trials including DAPA-CKD, EMPA-KIDNEY, CREDENCE, and CANVAS. Stages B and C focus on structural heart disease, both asymptomatic and symptomatic, with therapies targeting HFrEF and HFpEF, notably, DAPA-HF, EMPEROR-Reduced, EMPEROR-Preserved, and DELIVER. Additionally, these stages together address the prevention of worsening HF episodes and the transition to advanced HF.

The use of SGLT2i across the described phases is summarized in Figure 1 (Central Illustration).

## 4. Mechanisms of Action of SGLT2-Inhibitors

### 4.1. Direct and Indirect Pharmacological Action

The role of SGLT2i as cardio-renal protectors in both diabetic and non-diabetic patients is well-established; however, their precise mechanisms of action remain incompletely understood [59].

In HF pathophysiology, fluid retention and peripheral congestion activate the RAAS, leading to increased expression of SGLT2 receptors [37]. SGLT2i act by blocking the type 2 sodium-glucose cotransporter located in the S1 and S2 segments of the proximal convoluted tubule, which reabsorb approximately 90% of filtered glucose in the kidneys [6].

Additionally, SGLT2i target the sodium–hydrogen exchanger (NHE3), which operates synergistically with SGLT2, both functionally and structurally. NHE3 reabsorbs about two-thirds of the sodium filtered in the proximal nephron, playing a crucial role in fluid retention during heart failure while also contributing to glycemic homeostasis [38,52,60].

SGLT2i shift metabolism towards a catabolic state, increasing the glucagon-to-insulin ratio, promoting ketogenesis, and enhancing the utilization of free fatty acids (FFAs) and ketones as myocardial energy sources [61,62].

They decrease plasma volume through osmotic diuresis by inhibiting glucose and sodium reabsorption, primarily targeting interstitial congestion, unlike conventional diuretics that primarily reduce intravascular volume [63]. In addition to this osmotic diuresis, SGLT2i further enhance diuresis by disrupting the countercurrent multiplication system. This disruption reduces ion reabsorption in the loop of Henle, lowering the osmolarity of the medullary interstitium, which amplifies diuresis and contributes to the overall therapeutic effect [64]. The natriuretic effect of SGLT2i, although documented in several studies, remains an area of active discussion. An increase in sodium delivery to the distal nephron triggers compensatory mechanisms aimed at minimizing sodium loss. Evidence from a sub-analysis of the EMPA-RESPONSE AHF study suggests that empagliflozin, when used in patients with AHF, enhances plasma osmolarity without significantly affecting natriuresis, highlighting a nuanced interplay between SGLT2i and sodium handling in the kidney [65].

Beyond their debated natriuretic effect, SGLT2i exhibit significant renal protective properties. Elevated sodium concentrations in the distal nephron activate tubuloglomerular feedback, a key mechanism that mitigates hyperfiltration commonly observed in HF patients with heightened RAAS activation. This protective feedback loop sets SGLT2i apart from loop diuretics, which inhibit the NKCC transporter at the loop of Henle, reducing chloride delivery to the macula densa and inadvertently intensifying RAAS activation [52,66,67].

SGLT2i further counteract metabolic remodeling by enhancing hepatic ketone synthesis and reducing renal ketone excretion. Animal studies demonstrate that SGLT2i shift myocardial metabolism from anaerobic glycolysis to increased utilization of ketones, free fatty acids, and branched-chain amino acids. This metabolic reprogramming improves cardiac efficiency and reduces myocardial stress, contributing to the multifaceted therapeutic benefits of SGLT2i [68].

The initiation of SGLT2i therapy is associated with an initial decline in eGFR, primarily due to decreased intraglomerular pressure, a mechanism that varies across patient populations. In individuals with T2D, this effect is mediated by postglomerular vasodilation, while in type 1 diabetes (T1D), it results from preglomerular vasoconstriction driven by tubuloglomerular feedback [69,70,71,72,73]. Despite this early reduction in eGFR, long-term treatment with SGLT2i leads to improved renal function and slows the progression of CKD, highlighting their renal protective benefits [74,75].

### 4.2. Diuretic Action and Combined Effects with Other Diuretic Drugs

Considerable attention has been directed toward the potential diuretic effects of SGLT2i, in view of the importance of the concept of decongestion in HF [76,77,78,79]. The link between the clinical effects of SGLT2i and their decongestive properties first emerged in studies of cardiovascular outcomes in people with T2D [44,80]. In the RECEDE-CHF study, the addition of 25 mg of empagliflozin daily to loop diuretics resulted in increased urine production by about 500 mL on day three and sustained effects over six weeks in patients with HF and T2D [65]. Similarly, another trial involving high-dose loop diuretics showed that SGLT2i enhanced glycosuria and natriuresis, suggesting synergistic effects when combined with conventional diuretics [81]. Prospective studies further confirmed these findings, with one demonstrating a 1.5 L/day increase in diuresis within the first three days of SGLT2i initiation in hospitalized HF patients [82]. In the EMPIRE-HF study, a randomized, double-blind, placebo-controlled trial, stable patients with reduced ejection fraction were randomized to receive empagliflozin 10mg daily or placebo for 12 weeks. Treatment with empagliflozin resulted in a reduction in estimated extracellular volume, estimated plasma volume, and GFR at 12 weeks compared to placebo [83].

Studies exploring the interaction between SGLT2i and loop diuretics highlighted greater natriuresis when the two were combined [81,82]. However, variability in results across populations, dosing regimens, and interactions between different diuretics suggests the need for individualized treatment approaches. Despite these variations, SGLT2i consistently increased urine output and sodium excretion without causing hyponatremia, underscoring their safety and efficacy in managing congestion [37,84].

As reported earlier, EMPA-RESPONSE-AHF was the first study to provide robust evidence on the effects of SGLT2i in the setting of AHF [78]. In the study, patients assigned to empagliflozin showed an advantage in some indices of decongestion, with higher cumulative urine output up to day 4 compared to placebo. In the EMPAG-HF study, empagliflozin was associated with a 25% increase in cumulative urine output over 5 days when added to standard decongestant therapy, with an increase in diuretic efficacy [52]. By showing that SGLT2i increase daily urinary output and can therefore help to reduce the average dose of loop diuretics used in hospitalized patients [85], these results suggest a potentially important role for SGLT2i in the treatment of patients with AHF or with stable HF presenting with congestion early during the hospitalization in order to potentiate loop diuretics effects at lower dose, as shown.

### 4.3. Calcium Homeostasis

Calcium homeostasis is influenced by SGLT2i, extending their therapeutic effects beyond their diuretic and natriuretic actions. In HF and T2D, elevated levels of phospholamban impair the sarcoplasmic/endoplasmic reticulum calcium ATPase (SERCA2a) pump, which is vital for calcium ion reuptake at the end of systole. This impairment leads to diastolic dysfunction due to delayed calcium reuptake and systolic dysfunction from insufficient calcium availability for effective contractions [86]. Furthermore, increased phospholamban levels exacerbate diastolic dysfunction by enhancing ryanodine receptor (RyR2) phosphorylation, which reduces calcium storage within the sarcoplasmic reticulum [87]. Coupled with upregulated sodium–hydrogen exchanger 1 (NHE1) and SGLT1 expression, this results in elevated intracellular sodium and calcium concentrations, oxidative stress, and eventual cardiomyocyte death [61]. By enhancing SERCA2 function, reducing calcium loss from RyR2, and inhibiting NHE1 and SGLT1 expression, SGLT2i stabilize calcium dynamics and mitigate oxidative stress, restoring myocardial relaxation and contractility [63].

From a neurohormonal perspective, patients with HF and T2DM often experience disrupted calcium homeostasis and a metabolic shift driven by hypoxia. This shift leads to an increased reliance on glucose for ATP production as the primary energy source [88]. Prolonged exposure to such conditions results in metabolic remodeling, where cardiac cells develop a greater dependency on glucose [89]. Consequently, the accumulation of fatty acids, typically the preferred energy source for cardiac cells, causes lipotoxicity through the generation of reactive species and cellular steatosis, which ultimately contributes to cardiomyocyte dysfunction [90].

### 4.4. Hematological Effects

SGLT2i have been observed to correlate with increased hematocrit and erythropoiesis, an effect likely rooted in renal mechanisms [91]. This phenomenon is hypothesized to arise from overexpression of SGLT1 and SGLT2 in diabetic patients, leading to increased glucose uptake and heightened energy consumption, which induces cortical hypoxia. This state transforms fibroblasts into myofibroblasts, impairing their ability to produce erythropoietin (EPO) [92]. By reducing excessive energy demands and inhibiting SGLT1 and SGLT2, these drugs restore EPO production capacity. Additionally, SGLT2i enhances sodium delivery to distal nephron segments, upregulating sodium transporters in the loop of Henle and terminal nephron, which induces relative medullary hypoxia and further stimulates erythropoiesis [93].

### 4.5. Inflammation and Oxidative Stress

SGLT2i exhibit significant anti-inflammatory properties, effectively suppressing pro-inflammatory cytokines such as IL-1β and IL-18 through inhibition of the NLRP3 inflammasome in renal macrophages and cardiac fibroblasts [94,95,96]. Furthermore, they modulate multiple inflammatory pathways by reducing NF-κB activation, thereby lowering levels of markers like IL-6, TNF-α, monocyte chemoattractant protein-1 (MCP-1), and high-sensitivity C-reactive protein. These effects extend beyond cardiomyocytes, impacting noncardiac organs as well, contributing to their systemic anti-inflammatory benefits [97]. In terms of oxidative stress, SGLT2i help maintain nitric oxide (NO) homeostasis and mitigate oxidative damage by counteracting angiotensin II’s effects, inhibiting NADPH oxidase and cyclic guanosine monophosphate-dependent kinase (cGK-I), while promoting NO synthase phosphorylation [98,99].

Additionally, these drugs stabilize mitochondrial membranes and enhance energy production, which reduces vascular remodeling and microvascular dysfunction [97,100].

### 4.6. Cardiac Effects of Benefit

SGLT2i confer broad-ranging benefits to cardiac homeostasis through both direct and indirect mechanisms. Directly, they stabilize ion homeostasis, reduce oxidative stress, and function as anti-remodeling agents, notably, by mitigating cardiac fibrosis. Indirectly, they optimize preload and afterload dynamics, improve endothelial function, and lower oxygen demand through enhanced erythropoiesis. These effects collectively alleviate cardiac workload and improve systolic performance by reducing afterload [101].

Furthermore, iron homeostasis is essential for myocardial energy production, as iron-dependent enzymes facilitate mitochondrial oxidative phosphorylation [102]. In HF, iron deficiency is prevalent and exacerbates disease progression [89]. SGLT2i may counteract these effects by stimulating erythropoiesis through enhanced erythropoietin production and promoting iron mobilization [102]. Additionally, by modulating sodium–hydrogen exchanger activity and reducing oxidative stress, these agents improve mitochondrial efficiency, supporting iron-dependent enzymatic function and myocardial metabolism.

On the other side, diastolic function is shaped by two interconnected factors: the viscoelastic properties derived from the elasticity of myofilaments and extracellular matrix remodeling, as well as ion homeostasis, which governs the timing of muscle relaxation [103]. SGLT2i exhibit a dual advantage in this domain by stabilizing ionic balance and exerting anti-remodeling effects through the attenuation of systemic inflammation, a hallmark of HF. These combined actions lead to enhanced diastolic performance and a notable decrease in the arrhythmic burden commonly encountered in HF patients [6,97].

Furthermore, while large-scale trials have not reported a direct effect of SGLT2i on increasing heart rate (HR) via sympathetic system modulation, they have consistently demonstrated reductions in plasma levels of metanephrines and tyrosine hydroxylase, markers of sympathetic activity [104]. This suggests that SGLT2i can indirectly modulate sympathetic hyperactivation, a key contributor to ventricular remodeling and hypoxia observed in both HF and T2D [105,106]. This effect on the autonomic system is further amplified by the increased availability of ketone bodies, which suppress G-proteins in the sympathetic ganglia [107].

**Figure 2:**
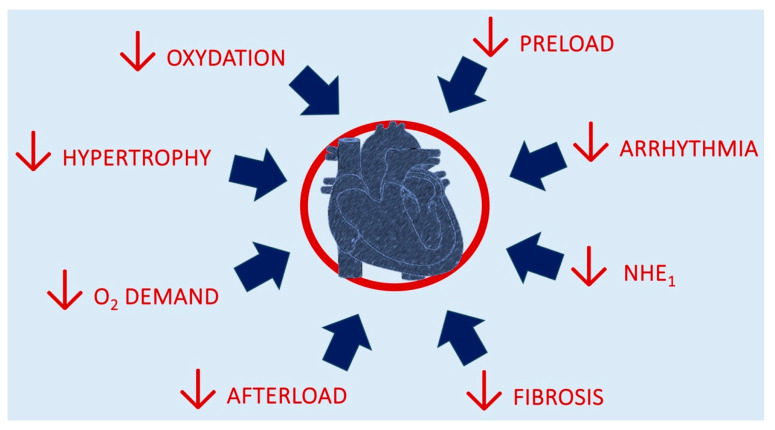
Mechanisms of putative benefits from SGLT2i introduction.

Figure 2 depicts the mechanisms for cardiac benefits from SGLT2i introduction.

## 5. Practical Considerations and Early Clinical Implementation

The integration of SGLT2i into HF management has been a transformative step, particularly given their demonstrated benefits across the spectrum of EF ranges [108]. While the clinical efficacy of SGLT2i is well established, practical considerations regarding their early implementation and real-world application warrant close attention.

### 5.1. Initiation and Titration in Heart Failure Therapy

Early initiation of SGLT2i is crucial in HF patients, irrespective of their LVEF [109,110]. In clinical practice, timely initiation and titration of HF therapies, including SGLT2i, are pivotal to maximizing their benefits [111]. The STRONG-HF trial highlighted that an intensive early up-titration strategy, within two weeks of hospital discharge, resulted in significantly reduced mortality and rehospitalization rates [112]. These findings underscore the importance of introducing SGLT2i promptly—ideally during hospitalization or immediately post discharge—alongside the other foundational therapies for HFrEF, ensuring therapy optimization in a controlled and monitored environment.

### 5.2. Safety and Tolerability Considerations

SGLT2i have demonstrated a favorable safety profile, but certain populations require specific considerations [113]. While these drugs are generally well tolerated, they are contraindicated in specific populations, including patients with T1D, those with a history of diabetic ketoacidosis, and those experiencing severe renal impairment [114,115]. Additionally, caution should be exercised when prescribing SGLT2i in patients with systolic blood pressure (SBP) lower than 95 mmHg or in those with conditions associated with significant volume depletion (e.g., gastroenteritis). For patients who present with reversible conditions like genitourinary infections, it may be necessary to temporarily withhold SGLT2i until the condition resolves. In elderly patients (≥75 years), SGLT2i are generally safe, but their use in very elderly patients (≥80 years) requires careful risk–benefit assessment, particularly due to the higher risk of volume depletion [116,117].

### 5.3. Impact on Blood Pressure and Diuretic Therapy

Post hoc analyses from major trials, such as DAPA-HF and EMPEROR-Reduced, have shown that SGLT2i cause a mild reduction in SBP, typically by 2–3 mmHg, without causing significant hypotension [116,118]. This reduction in SBP was observed across a range of baseline pressures, confirming the drug’s hemodynamic safety. Importantly, SGLT2i’s diuretic effect can complement traditional loop diuretics by enhancing decongestion, even in patients already on high-dose diuretics [119]. Unlike conventional diuretics, SGLT2i improve fluid management without exacerbating renal dysfunction, a common complication of loop diuretics [85]. These features make SGLT2i an invaluable addition to HF therapy, particularly in patients with comorbid renal dysfunction.

### 5.4. Special Considerations for Diabetic Patients

In patients with HF and concurrent T2D, glycemic control requires special attention [120]. Before initiating SGLT2i, clinicians should review existing antidiabetic regimens, particularly insulin or insulin secretagogues, as the combination may increase the risk of hypoglycemia; clinical guidelines recommend adjusting hypoglycemic agents if HbA1c levels are below 7.5% or if the patient has a history of recurrent hypoglycemia [121]. Additionally, SGLT2i reduce renal event risk, making them particularly valuable for patients with T2D, CKD, or at risk of contrast-induced acute kidney injury (CI-AKI) [122,123].

### 5.5. Practical Consideration in CKD Patients

Current ESC guidelines do not specify renal function monitoring or dose adjustments for minor eGFR declines observed upon SGLT2i initiation. Nevertheless, an increase in serum creatinine of less than 50 percent from the baseline value, provided that it remains below 266 µmol/L (3 mg/dL), or a decrease in eGFR of less than 10 percent from the baseline value, provided that eGFR remains above 25 mL/min/1.73 m^2^, can be regarded as an acceptable deviation, since in the long term, SGLT2i and other key drugs have been demonstrated to decelerate the progressive decline in eGFR, diminish proteinuria, and maintain renal function in comparison to placebo [124]. Consequently, these medications should not be discontinued without compelling justification.

### 5.6. Practical Implementation in the Hospital Setting

Real-world data support initiating SGLT2i during hospitalizations for AHF rather than waiting until discharge [53]. Initiating SGLT2i therapy during the hospital stay, as opposed to waiting until discharge, can significantly reduce the risk of future adverse events, including rehospitalization and death [37]. This reduces the risk of rehospitalization and mortality, allows for close monitoring of tolerability and side effects, and facilitates earlier diuretic synergy and enhanced decongestion. Moreover, the use of SGLT2i in the hospital setting, where patients are under close clinical observation, provides an opportunity to reinforce GDMT adherence post discharge. The EMPEROR and SOLOIST-WHF trials have demonstrated that initiating SGLT2i during the acute phase of HF results in better diuretic efficiency, earlier decongestion, and improved long-term outcomes without significant increases in adverse events such as hypotension or renal dysfunction [109,125].

### 5.7. Side Effects

Although essential agents in HF management, their use is associated with specific adverse effects requiring careful monitoring and punctual management. Among them, genital mycotic infections, primarily due to increased glucosuria fostering fungal overgrowth, occur in a variable 5–10% of patients, with a higher incidence among women and individuals with diabetes [126]. These infections are generally mild and can be managed with topical or oral antifungal therapy, while necessitating temporary discontinuation. Also, urinary tract infections (UTIs), initially a concern, have not demonstrated a significant increase in major trials, though real-world studies suggest an incidence of 2.4% at three months and 21.8% at six months [127]. Mild infections could be treated with standard antibiotics while continuing SGLT2i use, while recurrent or severe cases may warrant temporary discontinuation, although certain indications on this topic are lacking. Diabetic ketoacidosis (DKA), including euglycemic DKA, is a rare but serious complication occurring in approximately 0.1% of patients [128], particularly in insulin-dependent individuals or during acute illness or fasting. Preventive strategies include patient education; ketone monitoring in high-risk situations; and temporary discontinuation before major surgeries, prolonged fasting, or acute illness. In cases of suspected DKA, immediate withdrawal of SGLT2i, hydration, and insulin therapy are necessary. In addition, Fournier gangrene, a described possible side effect, is a type of necrotizing fasciitis or gangrene affecting the external genitalia or perineum. In the post-marketing surveillance, the Food and Drug Administration identified 55 cases in patients receiving SGLT2-inhibitors between 2013 and 2019 [129]. This infection is life threatening and requires immediate withdrawal, antibiotic treatment and surgical debridement. Furthermore, especially important in HF patients in high-dose diuretic therapy, volume depletion and hypotension can result from the osmotic diuretic effect of SGLT2i, particularly in elderly patients. Management includes ensuring adequate hydration; monitoring blood pressure; and, if symptomatic hypotension occurs, reducing or discontinuing concurrent diuretics rather than the SGLT2i itself. In severe cases, the drug may need to be temporarily held until fluid balance is restored. As presented earlier in the paper, acute kidney injury (AKI), although initially a concern due to transient declines in GFR of 3–5 mL/min in the first weeks [71], is usually reversible. SGLT2i should be continued in most cases, as long-term data suggest a renoprotective effect. Renal function should be closely monitored, and the drug should be temporarily discontinued in cases of severe AKI or significant hemodynamic instability. Some concerns also exist for lower limb amputation and bone fractures, reported specifically in the CANVAS trial with canagliflozin, which showed an increased risk of 6.3 amputations per 1000 patient-years and 15.4 fractures per 1000 patient-years, though these risks were not confirmed in other trials [45,47]. In high-risk patients with peripheral artery disease or osteoporosis, alternative agents such as empagliflozin or dapagliflozin may be preferred, with regular foot exams and bone health assessments to mitigate risk. Despite these potential adverse effects, the overall benefits of SGLT2i in reducing cardiovascular and renal events remain substantial. Individualized risk assessment, proactive monitoring, and patient education are key to optimizing therapeutic outcomes while minimizing adverse events. In most cases, side effects can be effectively managed without discontinuing SGLT2i therapy, ensuring patients continue to benefit from their cardioprotective and renoprotective effects.

### 5.8. Unmet Needs and Future Directions

In recent years, SGLT2i have emerged as promising agents in HF management, particularly in patients with both chronic and acute HF [34]. However, despite robust evidence supporting their use, several unmet needs and knowledge gaps persist, necessitating further investigation. One key area involves expanding our understanding of the role of SGLT2i in AHF. Trials such as EMPULSE and SOLOIST-WHF have demonstrated benefits in decongestion and reduced hospitalization rates in acute settings [28,69]. Nevertheless, more robust evidence is needed to establish the early use of SGLT2i as standard therapy during AHF episodes.

A critical knowledge gap lies in the optimal timing of SGLT2i initiation in AHF, especially when comparing patients with de novo AHF versus those with decompensated chronic HF [130]. While preliminary data and clinical reasoning favor early introduction, ongoing trials are expected to determine whether initiating SGLT2i immediately upon hospital admission—and maintaining treatment post discharge—offers additional benefits compared to the current practice of reserving SGLT2i for stabilized patients. Despite their clinical success, gaps remain regarding the long-term cardiovascular benefits of SGLT2i and the precise mechanisms underpinning these outcomes [131]. SGLT2i have demonstrated efficacy beyond glucose control, including renal function preservation, diuresis, and cardioprotection [113]. However, their molecular pathways of action remain incompletely understood [132]. Future research should aim to elucidate interactions between SGLT2i and key neurohormonal systems, such as the RAAS and the sympathetic nervous system, both of which are central to HF pathophysiology. While the diuretic and natriuretic properties of SGLT2i are well documented, their extent and mechanisms in promoting decongestion in acute settings require further investigation. A deeper understanding of how SGLT2i compare to traditional diuretics in efficacy, safety, and overall clinical outcomes could better inform their use in both acute and chronic HF scenarios.

## 6. Conclusions

SGLT2i have revolutionized the management of HF by demonstrating benefits across all stages of the disease. Uniquely, SGLT2i are the only class of drugs that effectively target the entire HF trajectory, from early prevention in high-risk individuals to the treatment of advanced HF, regardless of LVEF.

Major clinical trials, including DAPA-HF, EMPEROR-Reduced, and DELIVER, have consistently highlighted their significant impact on reducing hospitalizations, improving quality of life, and lowering cardiovascular mortality. These benefits extend across all LVEF phenotypes and are observed in patients with and without T2D and CKD, underscoring their broad safety, efficacy, and clinical applicability.

What distinguishes SGLT2i is their ability to address multiple pathophysiological mechanisms, including enhanced diuresis, renal protection, and improvements in cardiovascular outcomes. This versatility establishes SGLT2i as foundational of GDMT, proving especially valuable in both chronic and acute HF settings. Their demonstrated capacity to mitigate the risk of HF hospitalizations and cardiovascular death across the entire LVEF spectrum positions SGLT2i as indispensable agents in modern HF treatment paradigms.

Looking forward, the primary focus should be on optimizing early initiation of SGLT2i, particularly in acute HF settings, and expanding their use in emerging patient populations, such as those with advanced renal impairment or elderly individuals. Ongoing and future clinical trials are expected to further clarify their role and contribute to the refinement of clinical practice guidelines.

In conclusion, SGLT2i represent a transformative advancement in HF management, delivering comprehensive therapeutic benefits across all stages of the disease and fundamentally reshaping the treatment paradigm for this complex and heterogeneous condition.

## Figures and Tables

**Table 1:** Summary of current definitions in HF.

Heart Failure Classification	Definition
Definition	A clinical syndrome characterized by typical symptoms and/or signs due to a structural and/or functional cardiac abnormality, confirmed by elevated natriuretic peptides or objective evidence of congestion.
HFrEF	LVEF ≤ 40%
HFmrEF	LVEF 41–49%
HFpEF	LVEF ≥ 50%
HFimpEF	Previous LVEF ≤ 40% but now >40%
Worsening HF	Worsening signs or symptoms of HF requiring intensification of oral therapy, unplanned hospitalization, or IV diuretic administration, typically occurring within the chronic HF trajectory.

**Table 3:** Main available evidence for each stage of HF.

TRIAL	Study Design	Inclusion Criteria	Results and Conclusion
**STAGE A—PREVENTION**			
EMPA-REG-OUTCOME	7020 patients Empaglifozin + standard care vs. standard care	Patients with type 2 diabetes at high cardiovascular risk (previous MI, CAD, HF, stroke)	No significant differences in the rates of myocardial infarction or stroke, but the empagliflozin group presented significantly lower rates of death from cardiovascular causes (38% risk reduction), hospitalization for heart failure (35% risk reduction), and death from any cause (32% risk reduction)
DECLARE TIMI 58	17,150 patients Dapaglifozin vs. placebo	Patients with type 2 diabetes mellitus and either known cardiovascular disease or at least two risk factors for cardiovascular disease	Lower rate of cardiovascular death or hospitalization for heart failure (17% relative risk reduction) and showed a 24% risk reduction rate in renal events
CREDENCE TRIAL	4401 patients Canaglifozin vs. placebo	Patients with age ≥ 30 years, DM2, HbA1c of ≥6.5% and ≤12%, CKD (eGFR 30 to <90), urinary albumin-to-creatinine ratio > 300 to 5000 mg/g, stable dose of ACEi or ARB for ≥4 weeks before randomization	30% risk reduction for death from renal or cardiovascular causes and a 39% relative risk reduction for hospitalization for HF
CANVAS TRIAL	10,142 patients Canaglifozin vs. placebo	Patients with type 2 diabetes and high cardiovascular risk	The rate of death from cardiovascular causes, nonfatal myocardial infarction, or nonfatal stroke was lower with canagliflozin than with placebo (HR 0.86)
VERTIS CV TRIAL	8426 patients Ertuglifozin vs. placebo	Patients ≥ 40 years with T2DM diagnosis according to ADA guidelines and established ASCVD involving the coronary, cerebrovascular, and/or peripheral arterial systems	The occurrence of MACE was similar between the two groups, but lower rates of hospitalization for HF were seen in the ertugliflozin group than in the placebo group (8.1% vs. 9.1%)
SCORED TRIAL	10,584 patients Sotaglifozin vs. placebo	Patients with type 2 diabetes with HbA1c ≥ 7%, eGFR 25–60 mL/min/1.73 m^2^, CV risk factors	Lower risk of deaths from cardiovascular causes, and hospitalizations for heart failure, in the sotagliflozin group than in the control group, but higher rates of adverse events were experienced in the former group
**STAGE B–C—PRECLINICAL AND CLINICAL HF**			
DAPA-HF	4744 patients Dapaglifozin vs. placebo	NYHA class II–IV, with LVEF ≤ 40% despite OMT, and elevated NT-proBNP	The primary outcome occurred in 386 of 2373 patients (16.3%) in the dapagliflozin group and in 502 of 2371 patients (21.2%) in the placebo group. A first worsening heart failure event occurred in 237 patients in the dapagliflozin group and in 326 patients in the placebo group. Death from cardiovascular causes occurred in 227 patients in the dapagliflozin group and in 273 patients in the placebo group. Findings in patients with diabetes were similar to those in patients without diabetes.
EMPEROR REDUCED	1867 patients Empaglifozin vs. placebo	HFrEF patients, in NYHA class II–IV, and LVEF ≤ 40% despite OMT, and an elevated NT-proBNP	Empagliflozin was able to reduce the risk of CV death or HF hospitalization by 25% and to improve quality of life. This effect was consistent across patients with and without diabetes at baseline.
EMPEROR PRESERVED	5988 patients Empaglifozin vs. placebo	Patients with HF in NYHA class II–IV, whose LVEF was >40%, and elevated concentrations of serum NT-proBNP	Significant reduction of the primary endpoint, a composite of worsening HF and CV death (HR 0.79)
DELIVER TRIAL	6263 patients Dapaglifozin	Patients with HF in NYHA class II–IV, LVEF > 40%	Dapagliflozin reduced the primary endpoint of CV death or worsening HF by 18%
EMPULSE TRIAL	530 patients Empaglifozin vs. placebo	Patients hospitalized for acute de novo or decompensated chronic HF	Significant reduction in all-cause death as well as an improvement in quality of life 90 days after randomization
EMPA RESPONSE AHF	80 patients Empaglifozin vs. placebo	Patients with acute decompensated HF with or without diabetes	A post hoc analysis showed that SGLT2i had a synergistic effect, with loop diuretics leading to a higher urinary output and a more negative fluid balance in those treated with empagliflozin when compared with placebo
SOLOIST WHF	1222 patients Sotaglifozin vs. placebo	Patients with type 2 diabetes mellitus recently hospitalized for WHF	Significantly reduced cardiovascular deaths and HF hospitalizations (HR 0.67).
**STAGE D—ADVANCED HF**			
	Unfortunately limited data from large, randomized clinical trials that specifically address the management of medical treatment for this condition.		

Abbreviations: ACEi: Angiotensin-Converting Enzyme Inhibitor; ADA: American Diabetes Association; ARB: Angiotensin Receptor Blocker; ASCVD: Atherosclerotic Cardiovascular Disease; BMI: Body Mass Index; CAD: Coronary Artery Disease; CKD: Chronic Kidney Disease; CV: Cardiovascular; DM2: Type 2 Diabetes Mellitus; eGFR: Estimated Glomerular Filtration Rate; HbA1c: Glycated Hemoglobin; HF: Heart Failure; HFrEF: Heart Failure with Reduced Ejection Fraction; HR: Hazard Ratio; LVEF: Left Ventricular Ejection Fraction; MACE: Major Adverse Cardiovascular Events; MI: Myocardial Infarction; NT-proBNP: N-terminal prohormone of Brain Natriuretic Peptide; NYHA: New York Heart Association; OMT: Optimal Medical Therapy; SGLT2i: Sodium-glucose Cotransporter-2 Inhibitors; T2DM: Type 2 Diabetes Mellitus; WHF: Worsening Heart Failure.

**Table 4:** Data from cited studies for worsening HF.

TRIAL	Study Design	Inclusion Criteria	Primary Outcome	Secondary Outcome
EMPULSE [37]	530 patients Empaglifozin vs. placebo Median of F-UP: 3 months	Patient hospitalized for acute de novo or decompensated chronic HF	Composite of all-cause death, HF events, and in KCCQ-TSS using a win ratio that favored empaglifozin (1.36, [95% CI: 1.09–1.68]; *p* = 0.005)	Cardiovascular death or HF event Empagliflozin event rate: 55.01/100 patient-years Placebo event rate: 80.45/100 patient-years HR: 0.69 (95% CI: 0.45–1.08) Change from baseline in KCCQ-TS Empagliflozin: 36.19 vs. Placebo: 31.73
SOLOIST—WHF [38]	1222 patients Sotaglifozin vs. placebo Median of F-UP: 9 months	Patient with type 2 diabetes mellitus recently hospitalized for WHF	Total CV deaths, hospitalizations for HF, and urgent visits for HF Sotagliflozin event rate: 51.0/100 patient-years vs. Placebo event rate: 76.3/100 patient-years	Hospitalizations or urgent visits for HF: Sotagliflozin: 194 Placebo: 297/Rate ratio: 0.64 (95% CI: 0.49–0.83); *p* < 0.001 Cardiovascular death: Sotagliflozin: 51 Placebo: 58
VICTORIA [56]	5050 patients Vericiguat vs. placebo Median of F-UP: 11 months	Patient with chronic HF with EF < 45%; NYHA class II, III, IV; and evidence of WHF	Composite of CV death or first hospitalization for HF Vericiguat event rate: 33.6/100 patient-years Placebo event rate: 37.8/100 patient-years/HR: 0.90 (95% CI: 0.82–0.98); *p* = 0.02	Hospitalization for HF: Vericiguat event rate: 25.9/100 patient-years vs. Placebo event rate: 29.1/100 patient-years Cardiovascular death: Vericiguat event rate: 12.9/100 patient-years vs. Placebo event rate: 13.9/100 patient-years Total HF hospitalizations: Vericiguat: 1223 vs. Placebo: 1336

Abbreviations: ACEi: Angiotensin-Converting Enzyme Inhibitor; CI: Confidence Interval; CV: Cardiovascular; EF: Ejection Fraction; F-UP: Follow-Up; HF: Heart Failure; HR: Hazard Ratio; KCCQ-TSS: Kansas City Cardiomyopathy Questionnaire-Total Symptom Score; NYHA: New York Heart Association; WHF: Worsening Heart Failure.
